# Interactions of Cardiac Proteins with Plasma-Synthesized Polypyrrole (PSPy) to Improve Adult Cardiomyocytes Culture

**DOI:** 10.3390/polym16111470

**Published:** 2024-05-22

**Authors:** Teresa Gómez-Quintero, Roberto Olayo, Juan Morales-Corona, Omar E. Uribe-Juárez, César Millán-Pacheco, Rafael Godínez-Fernández, Iris N. Serratos

**Affiliations:** 1 Departamento de Ingeniería Eléctrica, Universidad Autónoma Metropolitana-Iztapalapa, Ciudad de México 09310, Mexico; tgomezq@xanum.uam.mx (T.G.-Q.); ouribe@xanum.uam.mx (O.E.U.-J.); 2 Departamento de Física, Universidad Autónoma Metropolitana-Iztapalapa, Ciudad de México 09310, Mexico; oagr@xanum.uam.mx (R.O.); jmor@xanum.uam.mx (J.M.-C.); 3 Facultad de Farmacia, Universidad Autónoma del Estado de Morelos, Cuernavaca 62209, Mexico; cmp@uaem.mx; 4 Departamento de Química, Universidad Autónoma Metropolitana-Iztapalapa, Ciudad de México 09310, Mexico

**Keywords:** plasma-synthesized polypyrrole, cardiac integrins, molecular docking, free energy determinations, culture of rat cardiomyocites

## Abstract

Plasma-Synthesized Polypyrrole (PSPy) has been reported as a biomaterial suitable for cell growth in vitro and in vivo. An experimental duplicate was carried out that showed the growth of cardiomyocytes with PSPy, following a protocol previously reported by the working group. The cardiomyocytes cultured with the biomaterial retained their native morphological characteristics, a fundamental key to improving cardiac cell therapy procedures. Such observations motivated us to investigate the molecular characteristics of the biomaterial and the type of interactions that could be occurring (mainly electrostatic, hydrogen bonds, and non-polar). Additionally, PSPy has been studied to establish the probable mechanisms of action of the biomaterial, in particular, its action on a group of cell membrane proteins, integrins, which we know participate in the adhesion of cells to the extracellular matrix, in adhesion between cells and as bidirectional signal transducer mechanisms. In this work, we carried out studies of the interactions established between cardiac integrins α2β1 and α5β1 with different PSPy models by molecular docking studies and binding free energies (ΔG_b_) calculations. The models based on a previously reported PSPy molecule have three variable terminal chemical groups, with the purpose of exploring the differences in the type of interaction that will be established by modifying the position of an amino (-NH_2_), a hydroxyl (-OH), and a nitrile (C≡N) in (fixed) groups, as well as the length of the terminal chains (a long/short -NH_2_). A model with short chains for the -OH and -NH_2_ (lateral) group was the model with the best interactions with cardiac integrins. We experimentally verified the direct interaction of cardiomyocytes with the PSPy biomaterial observed in rat primary cultures, allowing us to validate the favorable interactions predicted by the computational analysis.

## 1. Introduction

Cardiovascular diseases cause more deaths worldwide than any other type of non-communicable disease [1]. One-third of all deaths globally are due to cardiovascular diseases related to diseases of the heart or blood vessels, with myocardial infarction being one of the most common conditions [2,3,4]. Patients who have suffered considerable cardiac damage and who present with heart failure have few therapeutic options [5].

Different research groups within regenerative medicine and tissue engineering have focused on developing alternative therapies to heart transplantation for the restoration or attenuation of damage and recovery of the heart muscle [6,7]. Cell therapy is projected as one of the main alternatives for the restoration of cardiac function in situ [8]; however, problems related to the cell origin selected for the restoration, the low adhesion rate at the lesion site, the low survival and/or differentiation towards the correct lineages, the complexity of the native tissue, etc., remain to limit factors for restoration of cardiac function [9,10,11].

Within the research group, plasma-synthesized polypyrrole (PSPy) has emerged as a versatile biomaterial in in vitro and in vivo biomedical applications due to its electrical and morphological properties and richness of chemical surface. It has outstanding qualities as a scaffold or coating for materials used in cell cultures of various strains (hepatic cells, cartilage, nerve cells, bone tissue [12,13,14,15]). Regarding the application of PSPy in cardiac tissue engineering, in vitro studies showed that PSPy nanoparticles used as scaffolds for the primary culture of rat cardiomyocytes seem to prevent their cell dedifferentiation, in addition to providing adequate culture conditions for the preservation of cardiomyocytes for more than 30 days (a much longer viability period than in cultures without PSPy). It was also observed that the PSPy particles allowed primary cardiomyocytes to cluster in cell aggregates up to 1.2 mm^2^ with some aligned cardiomyocytes and a generation of fibers was observed on the surface of the aggregates (like the native extracellular matrix (ECM) of the tissue) [16]. Replicate experimental data and understanding the chemical characteristics of PSPy that may allow it to conform to cardiac microtissues and the possible formation of the surrounding extracellular matrix could elucidate the type of molecular interactions required in the biomaterial for better adhesion to cardiac tissues and its future application in in vivo models. In the longer term, it could provide new clues to improve the conformation of cardiac microtissues and apply them in cell therapy, where the extended proliferation and dedifferentiation promoted by PSPy materials would be useful for in situ repair.

It Is important to mention that the heart is composed of cardiomyocytes, fibroblasts, smooth muscle cells, endothelial cells, etc. The extracellular matrix components of the heart contain hyaluronic acid, fibronectin, proteoglycans, collagens, and laminins. Various mechanisms mediate the interaction of cardiac cells with the elements of the extracellular matrix. Still, one of the most important is through cell surface receptors such as integrins. In cardiac tissue, expressions of integrins α1β1, α2β1, α11β1, and β3 have been demonstrated in fibroblasts; meanwhile, expressions of integrins α1β1, α5β1, α7β1, and β1 have been demonstrated in cardiomyocytes [17].

Integrins constitute the main family of transmembrane proteins that interact with elements of the extracellular matrix, playing an active role in cell adhesion and migration processes or in signal transduction that regulates cell growth and differentiation. They are heterodimers made up of α and β units [18,19]. Some integrins have a metal ion-dependent adhesion site (MIDAS), at the center of which is a Mg^2+^ or Ca^2+^ ion and conserved amino acids in the surface loops. This MIDAS is located within the α domain of the integrin. The conserved amino acids in MIDAS and the coordination with the Mg^2+^ or Ca^2+^ ions are essential for the α subunit in binding with ligands of interest [20]. On the other hand, the β subunit recognizes different Arginine–Glycine–Aspartic Acid tripeptides (RGD motif) in the extracellular matrix (ECM) component structures [21].

**Figure 1:**
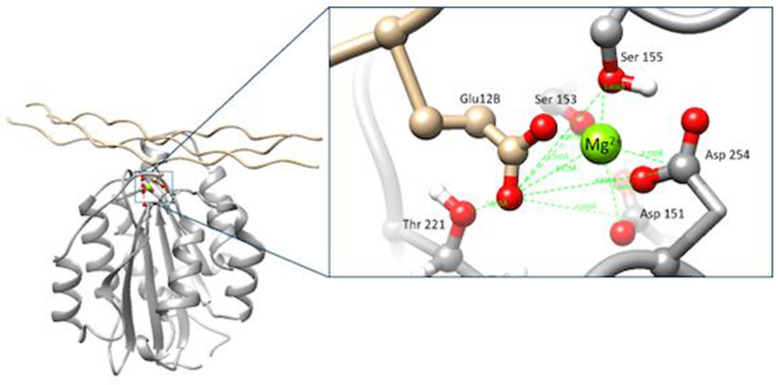
Overview of the interaction of the α2β1 integrin domain (PDB 1AOX) [23] and a collagen peptide (left; PBD 1Q7D) [25]; enlarged view of MIDAS and its interaction with glutamic acid in the GFOGER motif (right). Figures made with UCSF Chimera 1.15 software [26].

α2β1 integrin is a protein identified as an extracellular receptor that binds collagen and/or laminins [22]. The amino acids belonging to the MIDAS of α2β1 and that coordinate for the union of the integrin with collagen are Ser153, Ser155, Asp254, Asp151, and Thr221, either directly coordinating with the Mg^2+^ ion or indirectly through water molecules in an octahedral arrangement. The MIDAS structure recognizes a special motif present in native collagen fibers, the GFOGER (O: hydroxyproline) motif, present in different types of fibrillar collagen [23]. The GFOGER motif has high affinity with all reported collagen-binding integrins, although some integrins require conformational changes to obtain maximum binding. The interaction reported for the collagen peptide and the integrin domain is illustrated below [19,24], Figure 1.

**Figure 2:**
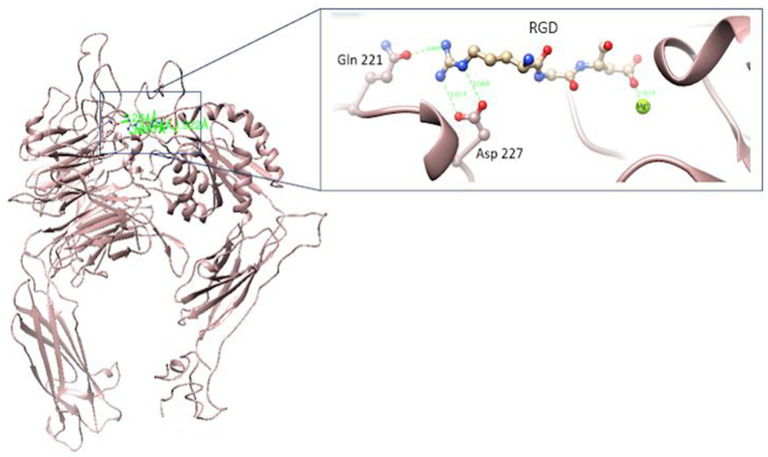
α5β1 integrin (PDB CODE 3VI4) interacting with RGD [28]. Figures made with UCSF Chimera 1.15 software [26].

The α5β1 integrin has a domain responsible for recognizing an RGD motif in fibronectin and fibrinogen molecules. The interaction of integrin and various extracellular ligands (fibrillin, VERGFR1, CD97, CD154, PHEV, etc.) depends on the structure of MIDAS, the presence of divalent cations, and their affinity for RGD motifs [27]. The amino acids of interest for the interaction of the integrin with the RGD motif are the amino acids Gln 221 and/or Asp 227 [28,29] (Figure 2).

**Figure 3:**
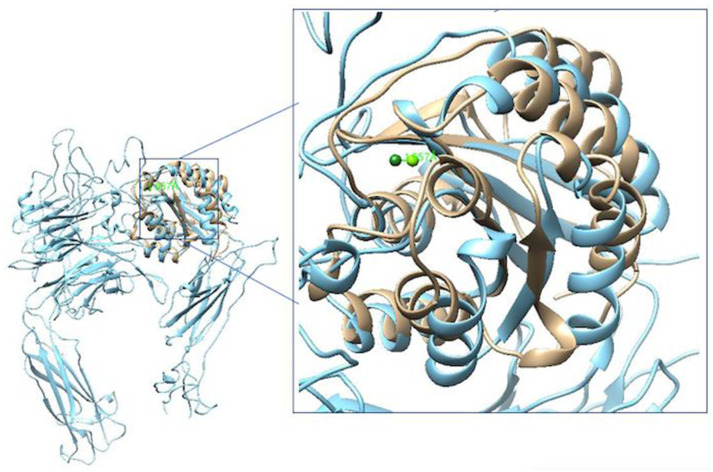
Comparison of the crystals of the minimized integrins 1AOX [23] (in cream color) and 3VI4 [28] (in blue color). There are considerable differences between both integrins domains. Its Mg^2+^ cations are out of phase by 1.657 Å. Figures made with UCSF Chimera 1.15 software [26].

The α2β1 and α5β1 integrin systems are found in cardiac tissue cells. Figure 3 shows the structural comparison of both domains. These structures will be used during the analysis by molecular docking of this work. The overlap of structures shows the similarities and differences between the two integrin domains.

**Figure 4:**
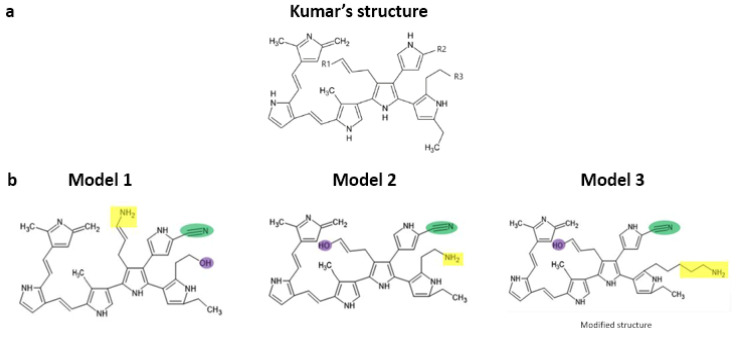
(**a**) The structure proposed by Kumar et al. [30], where the PSPy synthesized by plasma is shown. R1, R2, and R3 are terminal branches whose functional groups vary due to the richness of surface chemistry obtained by plasma synthesis. (**b**) The models used for this work; model 3 is a modified version of Kumar’s structure. Figure made in Python Molecule Viewer Version 1.3 [32].

Initially reproduced in a confirmatory manner, part of the main experimental results are reported by Uribe et al. [16]. We then proceed to a theoretical analysis of the possible molecular interactions of the PSPy biomaterial with the integrins of cardiac cells, which, as already mentioned, are important in cell adhesion and signaling. The starting hypothesis for the formation of cardiac microtissues is the interaction of components of the cell membrane and/or the extracellular matrix with surface chemical groups of the PSPy particles. In this work, computational modeling by molecular docking and binding free energy (ΔG_b_) calculations of the complexes formed by cardiac integrins (α2β1 and α5β1) and PSPy molecules with different terminal functional groups were performed. Two PSPy models are based on the structure reported by Kumar et al. [30] (Figure 4a), varying the position of -OH and -NH_2_ groups in R1 and R3, and leaving fixed the C≡N group in R2. A PSPy model was built and modified the R3 chain in the Kumar et al. structure, adding carbon atoms to make the chains longer (Figure 4b), to observe if the interaction is favored with integrins. Recently, Serratos et al. [31] used a modified structure and confirmed that when the -NH_2_ and -OH groups are in R1 and especially R3 positions of the PPPy structure, electrostatic interactions are strongly favored, then hydrogen bonds, and in minor proportion non-polar contacts in the interaction with albumin.

In our previous work, we carried out molecular docking assays and binding free energy (ΔG_b_) calculations of PSPy molecules with a collagen analogue peptide, as a first approach to modeling PSPy and its interaction with proteins that make up the extracellular matrix (ECM) as abundant as type I collagen. It was found that collagen interacts spontaneously in systems with PSPy; complexes with negative binding free energy were found for complexes with short chain PSPy, the amino group being the one that generated the most favorable interactions [33]. The data obtained from this first approach to modeling PSPy with structural biological proteins allowed us to make a new selection of proteins to perform a more exhaustive analysis of our biomaterial. The techniques of computational modeling by molecular docking and the determination of binding free energy are currently used in the design of biomaterials because it allows us to virtually assess the influence on the interaction of the material with a biological protein in a relatively simple way, which allows us to optimize experimental work times by modifying the characteristics of the biomaterial, the components for the synthesis, the culture conditions, etc.

Furthermore, the PSPy biomaterial plays a very important role in the tissue engineering. Molecular docking studies and the determination of binding free energy demonstrate the importance of the chemical diversity of the PSPy structure because it allows us to make combinations of functional groups that are present in experimentation such as the -NH_2_ and hydroxyl -OH groups, and these have been confirmed by IR studies in our research group [29,31]. These groups are of great influence in their interaction with proteins; hence, the interest of the study carried out in this work is that we experimentally verified the direct interaction of cardiomyocytes with the PSPy biomaterial observed in rat primary cultures. This allowed us to validate the favorable interactions predicted between cardiomyocytes integrins with PSPy structure by the computational analysis.

## 2. Materials and Methods

### 2.1. Culture of Adult Rat Cardiomyocytes

For the in vitro experiment with adult rat cardiomyocytes and PSPy particles, the methodology reported by Uribe et al. [16] was reproduced. The heart tissue of healthy animals was obtained from Dr. Gerardo Blancas Flores, who is part of the project “Farmacología y química de sustancias para el tratamiento del síndrome metabólico y otras enfermedades crónico-degenerativas” (code number: 1857) with the date of approval (31 January 2019). This project complies with the guidelines for the ethical conduct for Research, Teaching and Dissemination in the Comisión Académica de Ética of the División de Ciencias Biológicas y de la Salud of the Universidad Autónoma Metropolitana-Iztapalapa.

#### Cell Culture

The 1 × 10^4^ primary rat cardiomyocytes were seeded in 1 mL of M199 supplemented with 5% goat serum and 1% antibiotic-antimycotic; 200 μg of PSPy nanoparticles (nPSPy) were added to this cell suspension; the tube was centrifuged at 1200 rpm for 5 min (for two cycles, changing 1 mL of M199 on each occasion); and the final suspension was seeded in boxes of untreated culture of 35 × 100 mm. They were maintained under standard cell culture conditions, changing the medium every third day, carefully harvesting the cells with the nPSPy, and centrifuging to preserve the cells and material [16].

### 2.2. Plasma-Synthesized Polypyrrole Particles

**Figure 5:**
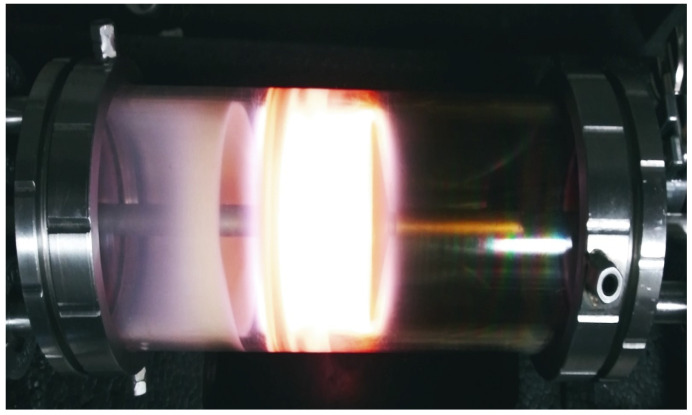
PSPy synthesis within a plasma reactor, with both electrodes separated by 5 cm, continues iodine doping.

PSPy particles were synthesized in a 12 cm long, 9 cm outer diameter, borosilicate cylindrical plasma reactor with 5 mm thick walls, with lateral disc electrodes with a diameter of 7 cm, separated 5 cm from each other. Electrodes are mounted in two stainless steel caps that close both sides of the cylindrical tube. The electrodes were connected to a 13.56 MHz radiofrequency source. The reactor was kept under vacuum for the synthesis, using polypyrrole monomer and continuous iodine (I) doping during the synthesis period, 1 h. The synthesis power was 45 W, with pressure inside the reactor of 1.6 Torr. The PSPy particles obtained had an average diameter, 330 ± 20 nm [16] (Figure 5).

### 2.3. SEM Micrographs of Cardiomyocytes Cultured with nPSPy

After an incubation period of 30 days, the biological samples were prepared for scanning electron microscopy through fixation and dehydration with alcohols, followed by a critical point drying process and the gold coating by sputtering. Analysis was performed in a JEOL 7600 HRSEM scanning electron microscope (JEOL Ltd., Tokyo, Japan).

### 2.4. Preparation of Structures

#### 2.4.1. Receptors

Integrin structures. Crystallized structures of the α2β1 (PBD ID 1AOX) [23] and α5β1 (PDB ID 3VI4) [25] domains, obtained from the Protein Data Bank, were minimized using CHARMM-GUI Version 3.8 to reduce stearic clashes, a 100-step Steepest Descent algorithm [34] as was described by Serratos et al. [31].

Notably, α5β1 integrin protein structure was simulated by 100 ns. Technical details for molecular simulation among trajectories can be found in Serratos et al. [31], and the cluster that obtained the best ΔG_b_ values was used for the molecular docking assays in the present work.

#### 2.4.2. Ligands

PSPy models. The PSPy ligands used for the molecular docking assays and free energy calculations were based on the structure proposed by Kumar et al. [30], varying the groups of the terminal chains R1, R2, and R3 with amino (-NH_2_), nitrile (-C≡N), and hydroxyl (-OH) groups; the carbon chain length of the amino group was modified for one of the PSPy models (to give the main chain better accessibility within the ligand). Ligands were designed in the PyMOL Molecular Graphics System Version 1.3, and structures were minimized in Avogadro Version 1.2.0 [32,35]. It is important to mention that the molecule proposed by Kumar et al. [30] is based on various characterization techniques and was proposed together with several functional groups that may or may not be in the R1, R2, and R3 chains; as it is a synthesis by plasma, it is difficult to determine a unique and exact structure of the components of the molecule. In our research group, several modeling works have already been carried out using the Kumar et al. molecule as a starting point for the modeling of PSPy interactions with various proteins [31,33,36]. The following ligands were designed:

- Model 1 has a -NH_2_ group in R1, a -C≡N group in R2, and R3 with a primary amine -OH.
- Model 2 has in R1 a -OH group, in R2 it has a -C≡N group, and in R3 -NH_2_.
- Model 3 has a -OH group in R1, a -C≡N group in R2, and R3 with a primary amine -NH_2_ accompanied by a 5-carbon aliphatic chain (-CH_2_- CH_2_-CH_2_-CH_2_-CH_2_-R3). The original structure proposed by Kumar et al. [30] only has a 2-carbon chain (-CH_2_-CH_2_-R3).

The lengthening of the terminal chain of the amino group was carried out to evaluate the effects of a more accessible amino group within the Kumar et al. molecule, taking advantage of the fact that aliphatic chains in solids are not very reactive and aliphatic chains are present in the spectra of the PSPy synthesized in the working group [29]. It was chosen for the terminal amino group because the study by Serratos et al. was one of the end groups with the best interactions in terms of binding free energy [31]. In the three models, R2 remained fixed with the nitrile group.

#### 2.4.3. Molecular Docking Studies

Molecular docking studies were performed on Autodock Vina implemented on PyMOL Molecular Graphics Systems Version 1.3 [37]. α5β1 integrin protein structure was simulated by 100 ns. Technical details for molecular simulation can be found in Serratos et al. [29]. The protein structure used for molecular docking as receptor is that obtained from clustering analysis. Each receptor–ligand system was made up of a target protein (α2β1 or α5β1) and a PSPy ligand (models 1, 2 or 3). Thus, it has six systems, see Table 1.

Around 400 poses were processed for each PSPy system and model. As the binding site for both integrin systems is already reported, the number of dockings per system was reduced. The interaction map was determined for each complex with LigPlot+ Version 2.2.4 software [38] and the binding energy was calculated (as described in the next section). Based on such maps and calculations, the best systems were chosen.

#### 2.4.4. Binding Free Energy Calculations for All Systems

PyMOL generates an output file in pdbqt format. This file consists of a modified pdb file that contains the atomic charges for the ligands, as well as topological information (bond rotations) [32]. These charges are used to for electrostatic calculations. The assignment of charges and radii for protein structures was performed with PDB2PQR Version 3.6.2 software [39]. However, the charges for the PSPy ligands were assigned by Autodock Vina Version 1.5.6rc2 [40]. To determine the electrostatic contribution of binding free energy, the methodology reported by Baker et al. [41]. The Baker method is a computational method for the determination of binding free energy (ΔG_b_) resulting from the electrostatic and non-polar interactions produced between receptors and ligands in biological systems (in continuous medium), Ec 1–2, where ΔG_elec_ is electrostatic free energy; ΔG_non-polar_ is the non-electrostatic or non-polar free energy; ΔG_sol_ is the solvation free energy; and ΔG_coul_ represents the coulomb component of the binding free energy. Coulombic and solvation contributions were calculated with Baker’s algorithm.

(1) $${∆G}_{b}={∆G}_{elec}+{∆G}_{non\text{-}polar};$$

where

(2) $${∆G}_{elec}={∆G}_{sol}+{∆G}_{coul}$$

The non-polar free energy was determined from an algorithm for calculating the solvent-accessible surface area (SASA) in the VMD Visual Molecular Dynamics (VMD) Version 1.9.4a53 software [42]. The ΔG_non-polar_ is proportional to the change in the area accessible to the solvent and a parameter similar to the surface tension (γ), with a value of de 0.021 kJ mol^−1^ Ǻ^−2^ [43]. With the numerical results obtained from the APBS [44] and VMD Version 1.9.4a53 [42] platforms from the pose files, the ΔG_b_ was calculated.

## 3. Results

### 3.1. Primary Culture of Cardiomyocytes with PSPy

**Figure 6:**
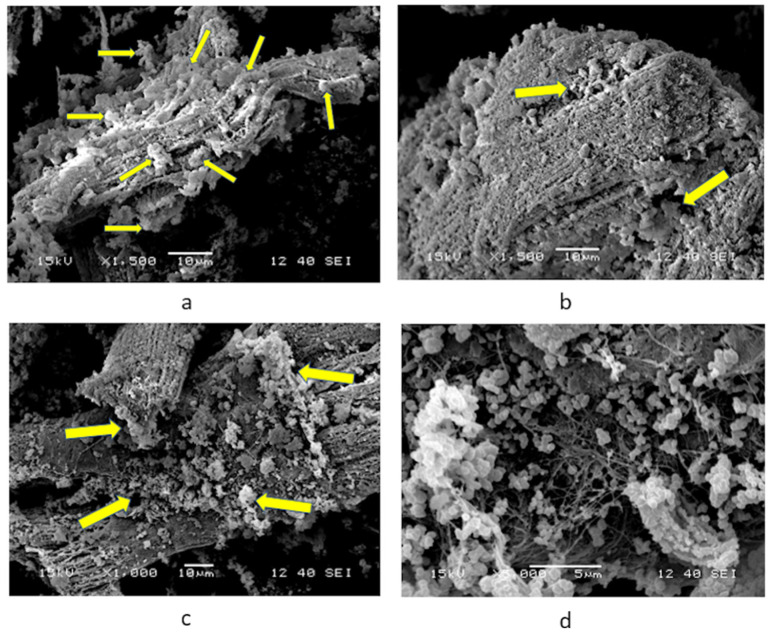
SEM microscopies for primary culture of cardiomyocytes with nPSPy. (**a**–**c**) Cardiomyocyte with particles, yellow arrows indicate clusters of nPPPy adhered to the adult rat cardiomyocyte (**a** and **b** ×1500, and **c** ×1000, scale 10 μm). (**d**) Greater detail of the PSPy particles and their interaction with fibrotic material belonging to the surface of the primary cardiomyocyte (×5000, scale 5 μm).

The results of the cell cultures of cardiac cells in the presence of PSPy were analogous to those reported by Uribe et al. [16], that is, the cells adhered to each other with the union of PSPy conglomerates on their surface, likewise generating fibrotic material that resembled extracellular matrix. The cultures lasted in optimal conditions until 30 days, the date on which the culture was stopped for the morphological analysis of the biological samples. At the end of the culture, the cells maintained their adhesion and characteristic morphology, that is, they maintained their differentiation (Figure 6). With these confirmatory results of those reported by Uribe et al. [16], the following question arises: What do PSPy do when interacting with the surface of cardiomyocytes that prolongs their life until they form microtissues in culture?

### 3.2. Interaction of the Integrin Domain with PSPy Ligand

**Figure 7:**
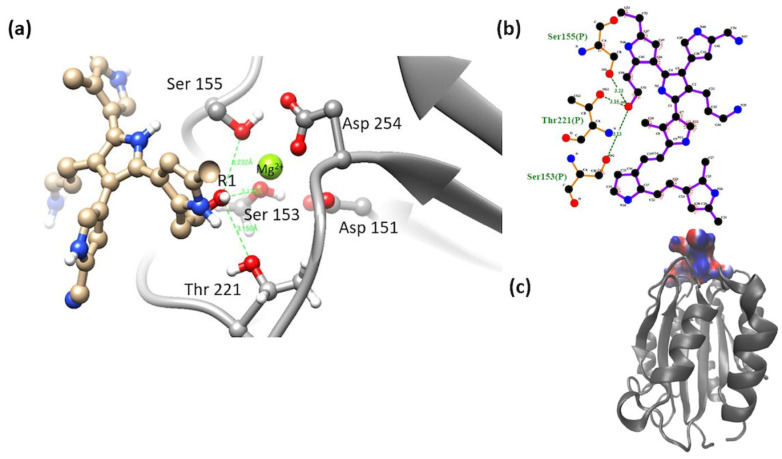
(**a**) MIDAS interaction of the α2β1 [23] integrin domain with PSPy model 1. Figures made with UCSF Chimera 1.15 software [26]. (**b**) A 2D interaction map made with LigPlot+ Version 2.2.4 software [38]. Hydrogen bonds are highlighted in green dotted lines. (**c**) Electrostatic potential of the PSPy model 1 in the binding site of the α2β1 integrin domain [23], where positive (blue color) and negative charges (red color) are shown. In minor proportion non-polar contacts are shown in white color. These figures were made in VMD Version 1.9.4a53 software [42].

α2β1-model 1. The system was unfavorable for this model, with a positive energy change (ΔG_b_ > 0). The interaction was mediated by the hydroxyl group in R3, on the side of the PSPy molecule, with some of the MIDAS elements not coordinating with the metal cation. The position of the OH group within the molecule is essential to establish an adequate interaction with the MIDAS elements. Loss of coordination with MIDAS elements produces weak interactions (non-polar contacts), hydrogen bonds, and electrostatic repulsion, which is why it obtained positive values for ΔG_b_; see Figure 7.

**Figure 8:**
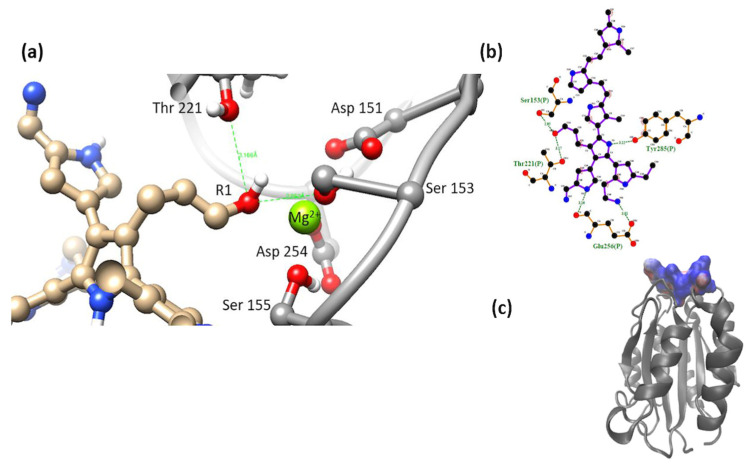
(**a**) MIDAS interaction of the α2β1 [23] integrin domain with PSPy model 2. Figures made with UCSF Chimera 1.15 software [26]. (**b**) A 2D interaction map made with LigPlot+ Version 2.2.4 software [38]. Hydrogen bonds are highlighted in green dotted lines. (**c**) Electrostatic potential of the PSPy model 2 in the binding site of the α2β1 integrin domain [23], where positive (blue color) and negative charges (red color) are shown. In minor proportion non-polar contacts are shown in white color. These figures were made in VMD Version 1.9.4a53 software [42].

α2β1-model 2. The most stable complex of this system showed interactions of MIDAS elements through the -OH group in R1 (mainly electrostatic and hydrogen bonds interactions, and non-polar weak interactions). The octahedral configuration reported between the MIDAS and collagen was not observed for this system [19], Figure 8. Despite the loss of coordination with the amino acids and metal cation of MIDAS, a much more negative binding free energy was calculated for this system, compared to the systems obtained for model 1. This may be due to the proximity of the nitrogens of the PSPy chains with the elements of MIDAS, in addition to the presence of interactions with other integrin amino acids (ΔG_b_ = −74 kJ/mol).

**Figure 9:**
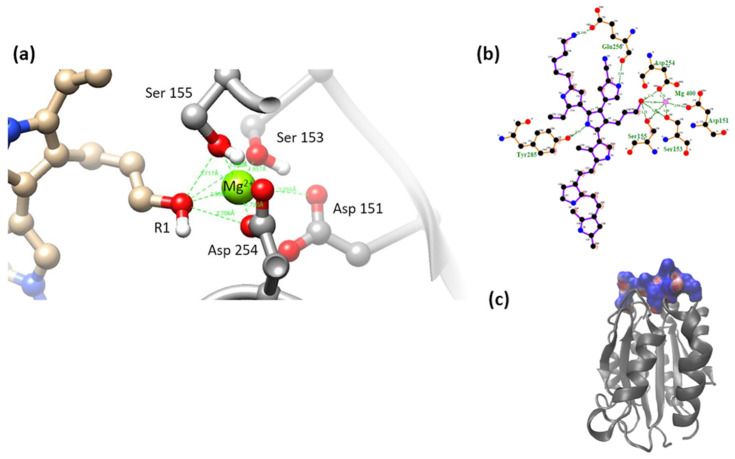
(**a**) MIDAS interaction of the α2β1 [23] integrin domain with PSPy model 3. Figures made with UCSF Chimera 1.15 software [26]. (**b**) A 2D interaction map made with LigPlot+ Version 2.2.4 software [38]. Hydrogen bonds are highlighted in green dotted lines. (**c**) Electrostatic potential of the PSPy model 3 in the binding site of the α2β1 integrin domain [23], where positive (blue color) and negative charges (red color) are shown. In minor proportion non-polar contacts are shown in white color. These figures were made in VMD Version 1.9.4a53 software [42].

α2β1-model 3. The most stable complex of this system showed that the hydroxyl group (-OH) located in R1 coordinated with some of the MIDAS amino acids (Ser 155, Ser 153, Asp 151, and Asp 254, including the Mg^2+^ ion). A semi-octahedral coordination similar to the reported configuration of the domain was observed with collagen [3] (Figure 9); the interactions of the integrin domain with the R1 group can occur due to the electrostatic attraction between the partial charges of the hydroxyl and those of the magnesium ion. These interactions were greatly benefited by the coulombic component hydrogen bonds and in minor proportion non-polar contacts in the binding, described in Table 2 (ΔG_b_ = −58 kJ/mol).

**Figure 10:**
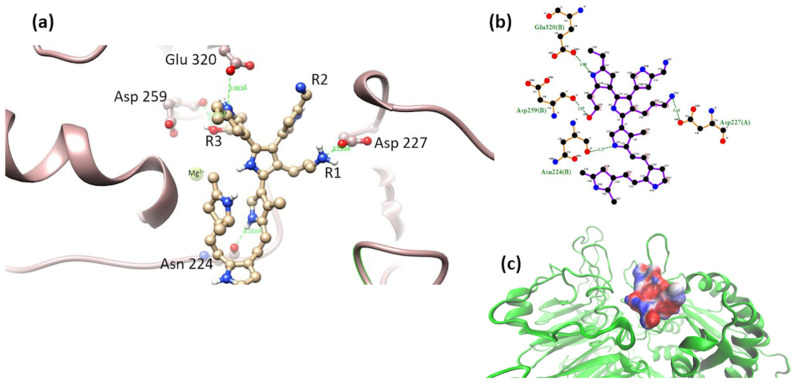
(**a**) MIDAS interaction of the α5β1 [28] integrin domain with PSPy model 1, Asp 227 interaction with PSPy model elements. Figures made with UCSF Chimera 1.15 software [26]. (**b**) A 2D interaction map made with LigPlot+ Version 2.2.4 software [38]. Hydrogen bonds are highlighted in green dotted lines. (**c**) Electrostatic potential of the PSPy model 1 in the binding site of the α5β1 integrin domain [28], where positive (blue color) and negative charges (red color) are shown. In minor proportion non-polar contacts are shown in white color. These figures were made in VMD Version 1.9.4a53 software [42].

α5β1-model 1. Interactions of the PSPy model with the short-chain R1 (-NH_2_) group were observed with Asp 227; the hydroxyl group in R3 (-OH) was shown to interact with the main chain of the amino acid Asp 259. This is in addition the interaction of the nitrogens of the PSPy rings with the main and side chain of the amino acids Glu 320 and Ala 260, respectively. Despite showing close interactions with the aforementioned amino acids, this complex obtained ΔG_b_ > 0. The coulombic free energy was almost positive and non-polar interactions and hydrogen bonds were slightly favorable (Figure 10).

**Figure 11:**
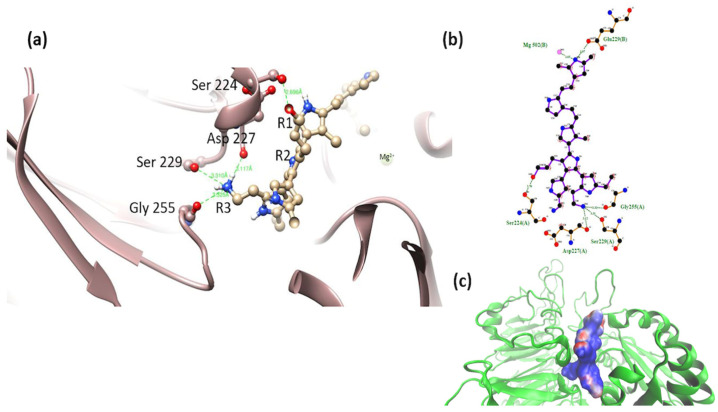
(**a**) MIDAS interaction of the α5β1 [28] integrin domain with PSPy model 2, the interaction of Asp 227 with PSPy model elements. Figures made with UCSF Chimera 1.15 software [26]. (**b**) A 2D interaction map made with LigPlot+ Version 2.2.4 software [38]. Hydrogen bonds are highlighted in green dotted lines. (**c**) Electrostatic potential of the PSPy model 2 in the binding site of the α5β1 integrin domain [28], where positive (blue color) and negative charges (red color) are shown. In minor proportion non-polar contacts are shown in white color. These figures were made in VMD Version 1.9.4a53 software [42].

α5β1-model 2. In this model, the -OH group-maintained interaction with a serine, and the short-chain -NH_2_ group interacted with Asp 227. PSPy maintained interactions with integrin through coordination with Mg^2+^ with nitrogen belonging to its rings (Figure 11). The binding free energy for this complex was negative (ΔG_b_ < 0), with a strong coulombic component due to the electrostatic interactions that were established at the binding site (ΔG_b_ = −331 kJ/mol) as well as hydrogen bonds and non-polar contacts.

**Figure 12:**
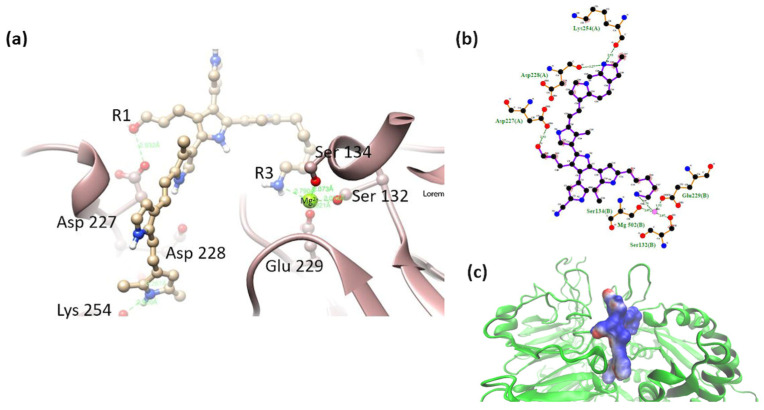
(**a**) MIDAS interaction of the α5β1 [28] integrin domain with PSPy model 3, the interaction of Asp 227 with PSPy model elements. Figures made with UCSF Chimera 1.15 software [26]. (**b**) A 2D interaction map made with LigPlot+ Version 2.2.4 software [38]. Hydrogen bonds are highlighted in green dotted lines. (**c**) Electrostatic potential of the PSPy model 3 in the binding site of the α5β1 integrin domain [28], where positive (blue color) and negative charges (red color) are shown. In minor proportion non-polar contacts are shown in white color. These figures were made in VMD Version 1.9.4a53 software [42].

α5β1-model 3. This model showed the interaction of the R1 (-OH) group with the amino acid Asp 227. Interaction with the MIDAS (including the metal cation Mg^2+^) of the integrin was maintained by coordinating with the amino group (-NH_2_) in R3 of the long side chain. It also interacted with nitrogens belonging to the PSPy rings (with the main chains of the amino acids Asp 228 and Lys 254). The electrostatic environment generated by the charges at the binding site produced a favorable interaction (ΔG_b_ < 0), as well as hydrogen bonds and non-polar contacts. Figure 12, ΔG_b_ = −247 kJ/mol.

**Figure 13:**
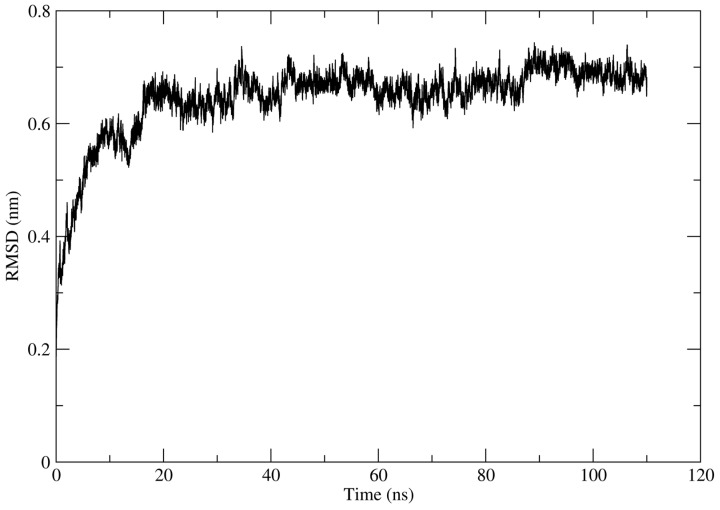
Evolution of RMSD over time of α5β1 integrin (PDB CODE 3VI4) [28].

After analyzing the interaction maps and determining values for the ΔG_b_ of the complexes formed, it can be observed that the system with the best interaction values corresponded to the α2β5 integrin. Therefore, a molecular dynamics analysis was performed to obtain a protein with conformational changes over time. The protein obtained was called α5β1 cluster1. Figure 13 shows the trajectory along 100 ns.

**Figure 14:**
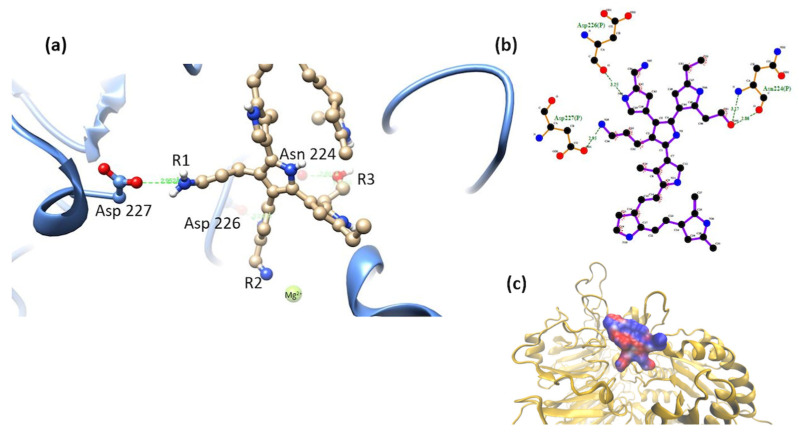
(**a**) MIDAS interaction of the α5β1 cluster-model 1 [28], Asp 227 interaction with PSPy model elements. Figures made with UCSF Chimera 1.15 software [26]. (**b**) A 2D interaction map made with LigPlot+ Version 2.2.4 software [38]. Hydrogen bonds are highlighted in green dotted lines. (**c**) Electrostatic potential of the PSPy model 1 in the binding site of the α5β1 integrin domain [28], where positive (blue color) and negative charges (red color) are shown. In minor proportion non-polar contacts are shown in white color. These figures were made in VMD Version 1.9.4a53 software [42].

α5β1 cluster-model 1. An interaction between the -NH_2_ group in R1 was preserved with the amino acid Asp 227. The R3 group (-OH) maintained interaction with the amino acid Asn 224 (main chain). The non-polar component strongly influenced the values of ΔG_b_ > 0 (Figure 14).

**Figure 15:**
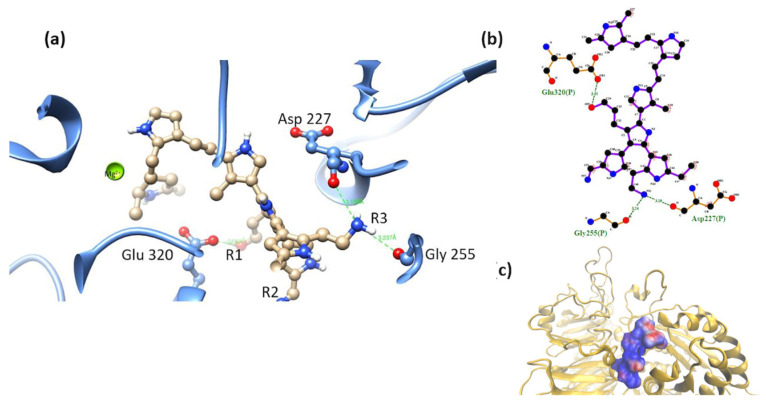
(**a**) MIDAS interaction of the α5β1 cluster-model 2 [28], the interaction of Asp 227 with PSPy model elements. Figures made with UCSF Chimera 1.15 software [26]. (**b**) A 2D interaction map made with LigPlot+ Version 2.2.4 software [38]. Hydrogen bonds are highlighted in green dotted lines. (**c**) Electrostatic potential of the PSPy model 2 in the binding site of the α5β1 integrin domain [28], where positive (blue color) and negative charges (red color) are shown. In minor proportion non-polar contacts are shown in white color. These figures were made in VMD Version 1.9.4a53 software [42].

α5β1 cluster-model 2. Interactions of the short-chain R3 (-NH_2_) group were observed with Asp 227 and Gly 255 (with backbone). The ΔG_b_ values were conserved within the same range before and after molecular dynamics. Coordination between PSPy and Mg^2+^ did not hold for these complexes after molecular dynamics. Figure 15, ΔG_b_ = −331 kJ/mol.

**Figure 16:**
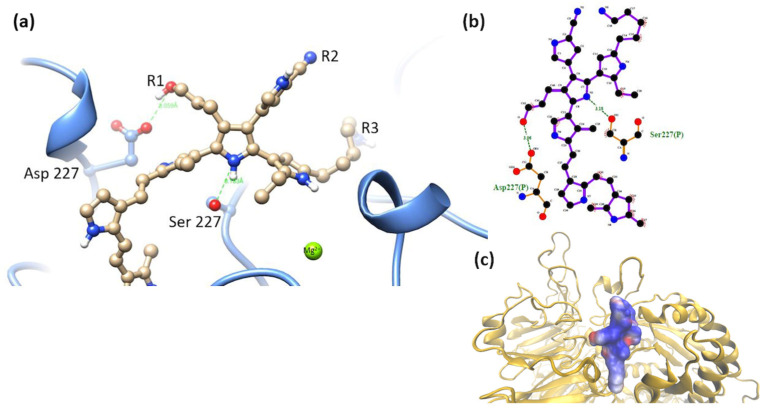
(**a**) MIDAS interaction of the α5β1 cluster-model 3 [28], the interaction of Asp 227 with PSPy model elements. Figures made with UCSF Chimera 1.15 software [26]. (**b**) A 2D interaction map made with LigPlot+ Version 2.2.4 software [38]. Hydrogen bonds are highlighted in green dotted lines. (**c**) Electrostatic potential of the PSPy model 3 in the binding site of the α5β1 integrin domain [28], where positive (blue color) and negative charges (red color) are shown. In minor proportion non-polar contacts are shown in white color. These figures were made in VMD Version 1.9.4a53 software [42].

α5β1 cluster-model 3. After molecular dynamics, the interaction with Asp 227 and -OH in R1 was preserved. The ΔG_b_ showed less favorable values (differences of up to 100 kJ/mol) but negative enough to define a spontaneous interaction despite having lost the coordination with the Mg^2+^ cation observed previously. Figure 16, ΔG_b_ = −89 kJ/mol.

It is important to mention that the present work aims to explain the experimental results reported by Uribe et al. [16], which has allowed us to know the molecular characteristics of the biomaterial in interaction with the integrins of cardiac cells. In addition, it has allowed us to obtain the most favorable interaction, which is a characteristic that should be preferentially obtained when manipulating the synthesis process. This cell type was chosen because the values for ΔG_b_ were shown to be much more favorable for this type of cardiac cells.

## 4. Discussion

### 4.1. Primary Culture of Cardiomyocytes with PSPy

The replicated experimental results based on the work of Uribe et al. [16] were the guideline for the computational analysis (the docking and the binding free energy calculations). In the SEM micrographs of the primary culture of rat cardiomyocytes with PSPy particles, an interaction between the cellular components and the biomaterial could be observed. The culture of these cardiomyocytes benefited from the interaction with the biomaterial, and the period of cell viability was extended for several weeks, in addition to the preservation of the typical morphology of the cardiomyocytes. PSPy seems to influence increasing cell viability and preserving the morphology of primary cultures of adult rat cardiomyocytes [16].

Molecular docking. For models 2 and 3, a preference for interactions mediated by the -OH group with MIDAS or elements of it was observed. The favored systems with a ΔG_b_ < 0 had a hydroxyl group at the R1 position. For mode 1, where the R1 group was replaced by the -NH_2_ group and the -OH group was placed in R3, we did not find favorable interactions, so it was found that MIDAS interacts with partially negative groups due to the action of electrostatic attraction of the magnesium cation (Mg^2+^). The molecular position of the group also influences whether or not complexes were obtained where favorable interactions could be observed; model 1 was an important example of the role of the terminal functional groups (R1 and R3) in the configuration of the ligand. The substituted group in R2, the nitrile group (C≡N), did not obtain an outstanding role for this system in any of the analyzed configurations. The length of the -NH_2_ group chains was changed in models 2 and 3 to assess whether a longer aliphatic chain could play an important role in the binding of the integrin domain to PSPy ligands. It was observed that the length of the chain of the amino group in model 3 did not favor the interactions; it was model 2 with the short-chain amino that obtained more favorable values for the interaction of the system.

In general, comparing the binding free energy values ΔG_b_ obtained for the PSPy ligands obtained for the interactions with the α2β1 and α5β1 integrins, we can observe that in the second system, α5β1, the binding free energy obtained much higher values than those calculated for the interactions with α2β1 for the second and third models. Model 1 (a molecule with R1: -NH2; R2: -C≡N; R3: -OH) obtained ΔGb > 0 values in both systems. The following table compares the values for the best complexes found for each integrin (two complexes per system).

Table 2 shows that systems with favorable ΔG_b_ were governed by the coulombic component and to a lesser extent by the non-polar component. Electrostatic interactions have a penalty for the desolvation of individual molecules, so the free energy of solvation was always observed to be positive [45,46].

In this work, with different models of the Kumar et al. [30] molecule, new values were found for the interactions of the biomaterial with the α5β1 integrin. Serratos et al. [29] modeled Kumar et al. [30] molecules with R1:R2:R3 with the same substituent functional group (same terminal group on each chain) and the same integrin. For the molecule determined by Kumar et al. with R1, R2, and R3 substituted with -NH_2_, Serratos et al. reported a ΔG_b_ = −447 kJ/mol, compared to the best value for our model 3 (ΔG_b_ = −247 kJ/mol). For the molecule substituted with three -OH groups, Serratos et al. [29] reported a ΔG_b_ = −87 kJ/mol, compared to the best value for our model 2 (ΔG_b_ = −331 kJ/mol). Finally, the positive values of the binding free energy for the interactions with the model 1 molecule (ΔG_b_ > 0) coincided with the positive values for the best complex of the Serratos et al. [29] molecules where the substituent of R1:R2:R3 was the nitrile group (-C≡N). In both systems, varying R1:R2:R3, the complexes obtained with values of ΔG_b_ < 0 showed such results because of the coulombic component (ΔG_coul_) and non-polar component (ΔG_non-polar_). From these first two systems and considering the values of ΔG_b_ as a reference, a molecular dynamics study was continued for the system with the best interaction values. Modeling the three PSPy models, the structure of α5β1 obtained from molecular dynamics will be called ‘α5β1 cluster’ to differentiate it from the minimized crystal. We were interested in whether favorable interactions with this integrin were preserved after configurational changes to the protein obtained from molecular dynamics.

### 4.2. Molecular Dynamics

**α5β1 cluster.** Table 3 compares the values for the best complexes found for the α5β1 integrin cluster (two complexes per system).

The ΔG_b_ for model 1 (where R1: short-chain -NH_2_, R2: -C≡N, R3: -OH) obtained positive values in the free energy calculations for the analyzed complexes (ΔG_b_ > 0). The interaction with the structural configuration of the PSPy of model 1 obtained unfavorable values of ΔG_b_. The interactions for the most stable complex formed with the minimized integrin showed interactions between R1(-NH_2_ short chain) with the amino acid Asp 227; the amino acid Asn 224 maintained close interaction with a nitrogen belonging to the PSPy rings. The interactions with the cluster showed that the R1 (-NH_2_) group had a close interaction with Asp 227; the R3 group (-OH) maintained an interaction with Asn 224. However, the calculated values of ΔG_b_ were positive; it was the ΔG_sol_ component that had the greatest contribution for the determination of the binding free energy.

Model 2 (where R1: -OH, R2: -C≡N, R3: short-chain -NH_2_) preserved the range of values for ΔG_b_ obtained for the best complexes before and after molecular dynamics. The interaction with the structural configuration of the PSPy of model 2 obtained favorable ΔG_b_ values within the same range for the complexes before and after carrying out the molecular dynamics. Before molecular dynamics, the complex with the best ΔG_b_ showed the coordination of Mg^2+^ with nitrogen belonging to the PSPy rings. The R3 group (-NH_2_ short chain) maintained interaction with Asp 227 and other amino acids; the R1 (-OH) group showed interaction with Ser 224. ΔG_coul_ is the component with the greatest contribution to the formation of interactions (electrostatic, hydrogen bonds and in minor proportion non-polar contacts) of this complex, which agrees with other systems [47,48,49,50].

It is noteworthy that the binding between a protein and a ligand involves non-covalent interactions such as electrostatic interactions, hydrogen bonds, van der Waals interactions, hydrophobic interactions, and π-cation interactions [51,52]. In this work, we validated these interactions.

Mostly, comparing the ΔG_b_ values obtained for the most favorable complexes in each model, before and after performing molecular dynamics on the protein, a decrease in the binding free energy values can be observed for the complexes of model 3 (where R1: -OH, R2: -C≡N, R3: long-chain -NH_2_). The interaction with the PSPy structural configuration of model 3 was favorable for interactions between the integrin and the PSPy ligand to be generated. Before molecular dynamics, the long chain amino group directly coordinated with the Mg^2+^ cation; interaction between the hydroxyl group with the side chain of the amino acid of interest Asp 227 and other interactions with nitrogens belonging to the PSPy rings were also established. After performing molecular dynamics, the coordination with Mg^2+^ was lost, but the interaction of the Asp 227 side chain with the hydroxyl group at the center of the PSPy molecule was preserved. ΔG_coul_ is the component with the greatest contribution to the formation of interactions of this complex.

In all three models, the interaction with Asp 227, essential for native integrin binding, was preserved; however, the coordination with the magnesium cation was not maintained.

**Figure 17:**
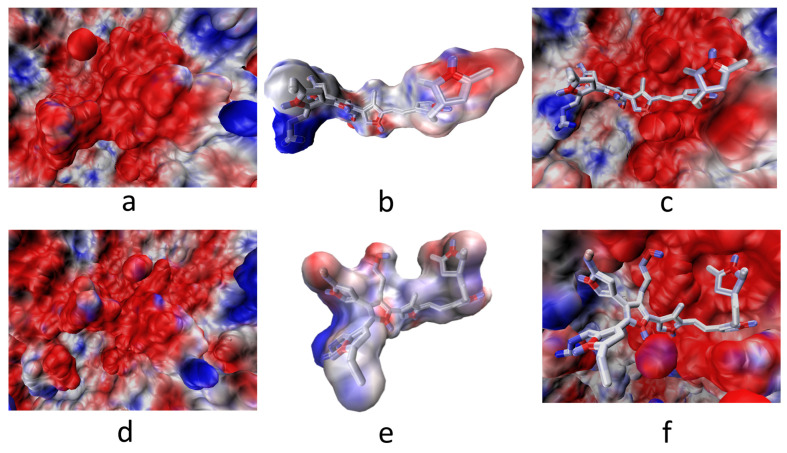
Electrostatic potential maps for PSPy model 2 and the α5β1 integrin before and after dynamics. (**a**) Minimized α5β1. (**b**) PSPy model 2. (**c**) Minimized integrin complex. (**d**) Clustered α5β1. (**e**) PSPy model 2. (**f**) Clustered integrin complex. In blue, the areas with positive charges are shown, in red the negative ones, in white non-polar contacts. In both systems, the integrin binding site is predominantly negatively charged; after binding with the PSPy ligand, the charge variation is observed when such complexes are formed. These figures were made in VMD Version 1.9.4a53 software [42].

Finally, model 2 of PSPy with the integrin α5β1 cluster being the system that best conserved its affinity concerning its binding free energy values (ΔG_b_) shows the electrostatic potential maps of the interaction below. It should be remembered that the coulombic component of the systems was precisely the predominant interaction that led to the spontaneity of the interactions found (red and blue color) and non-polar contacts (in white) (Figure 17).

## 5. Conclusions

This type of study allows us to know the preferential surface conditions of biomaterials to interact favorably with biological systems such as cardiac cells. As can be seen from this study, some PSPy configurations with their functional groups present a more favorable interaction with a type of integrin and even with some of its subunits, while others are less favorable to interact with integrins.

Model 1, where R1 was replaced by the -NH_2_ with a short chain and R3 by the -OH, was not favorable for the complexes analyzed for any integrin, neither before nor after minimization and dynamics (for the model of the α5β1 cluster).

The systems analyzed for the PSPy ligands showed favorable interactions (ΔG_b_ < 0), for both cardiac integrins, α2β1 and α5β1 (minimized and clustered). Favorable interactions were preserved for the models in which the PSPy had the central -OH in R1 and the lateral -NH_2_ in R3 (long and short chain). It was model 2 that maintained the best binding free energy favors for the minimized integrins and the α5β1 cluster.

The configuration of the terminal groups in the Kumar et al. [30] molecule, R1, R2, and R3, play a fundamental role in the type of interaction and binding free energy values obtained for complexes with cardiac integrins. The terminal amino and hydroxyl groups were shown to be suitable for forming favorable complexes with cardiac integrins, the short-chain amino group being favored and in a lateral position within the Kumar’s molecule.

Regarding the in vitro experiment of the adult cardiomyocytes from the primary culture, it was possible to verify the direct interaction of the cells with the PSPy. The nature of the interaction is still unknown, but the results of the computational analysis allow us to propose favorable interactions due to the presence of groups -OH and -NH_2_ in the terminal chains of the PSPy. The ability of PSPy to maintain interactions with cardiac cells in systems like native interactions (with RGD) could benefit the growth and preservation of morphologies in cultured cardiomyocytes.

The analysis by molecular docking, molecular dynamics, and the determination of free binding energy in cardiac systems was fundamental for the determination of possible interactions between ligands of the biomaterial and cardiac cell cultures. Having more information about the possible favorable interactions that the biomaterial can have within the biological system will allow us to determine if the ligand of interest could be a good cellular scaffold based on the molecular interactions that could be generated or not with cellular integrins.

## Figures and Tables

**Table 1:** Groups analyzed through molecular docking, two receptors, and three cases (three different configurations for the PSPy molecule).

Systems		PSPy Ligands		
		Model 1	Model 2	Model 3
Receptors	α2β1	α2β1-model 1	α2β1-model 2	α2β1-model 3
	α5β1	α5β1-model 1	α5β1-model 2	α5β1-model 3

**Table 2:** Comparison of binding free energy (ΔG_b_) for models of PSPy and integrins α2β1 and α5β1.

Ligand	Integrin	System	ΔG_sol_ (kJ/mol)	ΔG_coul_ (kJ/mol)	ΔG_non-polar_ (kJ/mol)	ΔG_b_ (kJ/mol)
Model 1	α2β1	C1	85	−18	−21	>0
		C2	91	−14	−20	>0
	α5β1	C1	87	1	−21	>0
		C2	98	−1	−24	>0
Model 2	α2β1	C1	81	−136	−19	−74
		C2	82	−138	−18	−74
	α5β1	C1	115	−421	−25	−331
		C2	125	−425	−25	−325
Model 3	α2β1	C1	92	−128	−22	−58
		C2	98	−130	−21	−53
	α5β1	C1	149	−370	−26	−247
		C2	188	−382	−25	−219

C1: Complex 1, C2: Complex 2, ΔG_sol_: Solvation free energy, ΔG_coul_: Coulombic free energy, ΔG_non-polar_: Non-polar free energy, ΔG_b_: Binding free energy. Only C1 for each complex was explained in the Section 3.

**Table 3:** Comparison of binding free energy (ΔG_b_) for models of PSPy and integrins α2β1 and α5β1. The values of α2β1 are shown in blue, in red the values for the integrin α5β1.

Ligand	Integrin	System	ΔG_sol_ (kJ/mol)	ΔG_coul_ (kJ/mol)	ΔG_non-polar_ (kJ/mol)	ΔG_b_ (kJ/mol)
Model 1	α5β1	C1	87	1	−21	>0
		C2	98	−1	−24	>0
	α5β1 cluster	C1	119	2	−21	>0
		C2	166	−18	−26	>0
Model 2	α5β1	C1	115	−421	−25	−331
		C2	125	−425	−25	−325
	α5β1 cluster	C1	184	−482	−23	−321
		C2	135	−313	−21	−199
Model 3	α5β1	C1	149	−370	−26	−247
		C2	188	−382	−25	−219
	α5β1 cluster	C1	236	−299	−26	−89
		C2	253	−312	−27	−86

C1: Complex 1, C2: Complex 2, ΔG_sol_: Solvation free energy, ΔG_coul_: Coulombic free energy, ΔG_non-polar_: Non-polar free energy, ΔG_b_: Binding free energy. Only C1 for each complex was explained in the Section 3.

## Data Availability

For the structural dataset used reported in this article are available in the Protein Data Bank (PDB): Crystal structure of the I domain from integrin alpha2beta1 from Homo sapiens. “https://www.rcsb.org/structure/1aox (accessed on 4 December 2023)”. Crystal structure of alpha5beta1 integrin ectodomain: Atomic details of the fibronectin receptor from Homo sapiens. “https://www.rcsb.org/structure/3vi4 (accessed on 4 December 2023)”. Structure of the Integrin alpha2beta1-binding Collagen Peptide from Homo sapiens. “https://www.rcsb.org/structure/1q7d (accessed on 4 December 2023)”. The datasets generated and/or analyzed during the current study are available in the Zenodo repository. “https://doi.org/10.5281/zenodo.10342011 (accessed on 10 December 2023)”.
